# Double-lumen endobronchial tube in the emergency management of massive hemoptysis

**DOI:** 10.4103/0974-2700.66527

**Published:** 2010

**Authors:** Luciano Santana-Cabrera, Manuela Fernández Arroyo, Alina Uriarte Rodriguez, Manuel Sanchez-Palacios

**Affiliations:** Intensive Care Unit, Universitary Hospital Insular in Gran Canaria, Las Palmas de Gran Canaria, Spain

Sir,

Massive hemoptysis (600 ml in 24 h) results in a mortality of more than 50%.[1] The cause of death is usually asphyxiation rather than exsanguination during the first hour. Independent lung ventilation is often used intraoperatively but it has also been used in a variety of critical situations when the lung abnormality is predominantly unilateral.[2] The correct use of this technique can keep the patient alive till definitive treatment – endovascular or surgical – is available.

We report the case of a 53-year-old man with a history of pulmonary tuberculosis who was admitted to the emergency department after a cardiorespiratory arrest secondary to massive hemoptysis. After resucitation and stabilization of the patient, significant bleeding continued through the endotracheal tube. Bronchoscopy was performed to locate the source and showed that the bleeding was from the upper right lobe. Due to the significant amount of blood in the bronchial tree, which made ventilation of the patient difficult, we decided to carry out selective intubation of the left bronchus with a double-lumen tube so as to achieve adequate ventilation and oxygenation. After the intubation, correct placement of the tube was checked with bronchoscopy and the patient was transfered to the radiology room where embolization of a pathological vascular lesion that was supplied by the right bronchial artery was performed. The bleeding eventually stopped.

This report calls attention to the fact that, until the completion of definitive treatment, independent lung ventilation is an option to be considered in cases of unilateral pulmonary abnormalities when conventional mechanical ventilation is likely to be deleterious for the patient.

Bronchial artery embolization is a safe and effective palliative treatment alternative in moderate and massive haemoptysis.[3,4] Thoracotomy with double-lumen endotracheal intubation and resection of the cavity may be curative and lifesaving when other measures fail in the management of tuberculosis-related hemoptysis; the problem is that many patients are unfit for surgery because of the acute hypoxemia and limited lung capacity.[5]

Selective intubation is a procedure to be carefully performed and should only be done by those with complete understanding of the symptoms and signs of tracheobronchial tree injuries.[6] There are clinicians who achieve correct positioning of a double-lumen endobronchial tube without the aid of any instruments. It is not a time-consuming technique and can be taught easily. The method may be vital in a situation where rapid lung isolation or collapse is the key to saving life. This blind method can be an alternative to the fiberoptic bronchoscope or auscultation for the positioning of double-lumen tube in an emergency situation.[7]
